# To Be in Pain: Pain Multidimensional Questionnaire as Reliable Tool to Evaluate Multifaceted Aspects of Pain

**DOI:** 10.3390/jcm13195886

**Published:** 2024-10-02

**Authors:** Giuseppe Forte, Francesca Favieri, Vilfredo De Pascalis, Maria Casagrande

**Affiliations:** 1 Department of Dynamic and Clinical Psychology and Heath Studies, “Sapienza” University of Rome, 00185 Rome, Italy; g.forte@uniroma1.it (G.F.); maria.casagrande@uniroma1.it (M.C.); 2 Department of Psychology, “Sapienza” University of Rome, 00185 Rome, Italy; vilfredo.depascalis@uniroma1.it

**Keywords:** pain, questionnaire, validation, pain sensitiveness, factorial structure

## Abstract

**Background/Objectives:** Pain is a multidimensional experience influenced by sensory, emotional, and cognitive factors. Traditional pain assessments often fail to capture this complexity. This study aimed to develop and validate the Pain Multidimensional Questionnaire (Pa-M-QU), a new self-report tool designed to assess pain catastrophizing, sensitivity, and coping strategies. **Methods:** Two independent samples of Italian-speaking participants, aged 18 and above, were recruited online. The first sample (*n* = 392; mean age = 29.36) was used for exploratory factor analysis (EFA), and the second sample (*n* = 123; mean age = 28.0) for confirmatory factor analysis (CFA). Pearson’s correlations and convergent validity analyses were conducted. **Results:** From an initial pool of 59 items identified through focus group discussions, 35 items were removed based on reliability analysis. The final 24-item Pa-M-QU features a three-factor structure: catastrophizing, pain sensitivity, and coping with pain. **Conclusions:** The Pa-M-QU offers a rapid, non-invasive assessment that captures the multidimensional nature of pain. It is a starting point to develop tools for both clinical and research settings, aiding in evaluating pain in healthy individuals and predicting acute and chronic pain disorders. Future research should focus on refining the Pa-M-QU for broader clinical applications and exploring its potential to complement or replace traditional pain assessments, thereby advancing pain management and research.

## 1. Introduction

*“I focus on the pain. The only thing that’s real”.*

Johnny Cash

Since its foundation, the International Association for the Study of Pain (IASP) has been faced with the challenge of establishing definitions of pain that needed simultaneously to align with the significant advances in the scientific basis of nociception and to be pragmatic for individuals experiencing a spectrum of pain conditions, from acute to chronic [1]. A first definition considered pain as “an unpleasant sensory and emotional experience associated with actual or potential tissue damage or described in terms of such damage” [1]. However, understanding and explaining the experience of pain is a challenging endeavor, especially when considering its verbal communication [2]. As suggested by Cohen and colleagues, individuals experiencing pain have no direct language in which to express that experience to themselves and others [3]. Consequently, they tend to rely on metaphorical and comparative language. This linguistic issue also arises in clinical practice where, for oversimplification, pain is commonly defined as a correlation between organic damage and pain reported by the patients. This definition excludes cases where there is pain without evident tissue damage. Pain is a more complex phenomenon than the relationship with damage. Pain can be considered, in its nature and intensity, a critical component of conscious experience, affecting thoughts, emotions, and overall mental state. Accordingly, a recent theorization considers pain as a personal distressing experience characterized by multiple sensory, emotional, and cognitive components, which are associated with actual or potential damage [4,5]. In this sense, the subjective experience of pain is relevant because it highlights the existence of interindividual variability in pain perception, even in the case of similar injuries. Furthermore, it marks the influence of biological, psychological, and social factors on pain experience [6].

In this context, the concept of pain sensitivity became central. The substantial interindividual variability in pain sensitivity [7] has received increasing attention in recent years [8]. For example, higher sensitivity was observed in experimental models of induced pain in individuals with chronic pain disorders (e.g., chronic tension-type headache, fibromyalgia, temporomandibular dysfunction, and chronic low back pain), which raises the question of whether the response to an acute nociceptive stimulation may indicate a predisposition to develop a chronic pain disorder or if the reported pain sensitivity in an experimental setting increases during the course of the disease [9]. The assessment of pain sensitivity and its components is of crucial importance in the context of clinical pain conditions. Several tools have been developed for the evaluation of these aspects in conditions beyond the experimental setting. This aspect is relevant because a manipulated setting, such as that of an experimental model, allows for the indirect evaluation of pain perception and sensitivity, which may not fully reflect the ecological and real-life components [10]. Moreover, sensitivity is only one aspect that should be considered. For example, negative cognition related to the experienced pain (i.e., pain catastrophizing) as well as the ability to manage pain would provide a more comprehensive framework for understanding pain experience, given their close relationship to both the perception of the experience and the therapeutic outcomes. These aspects, which have been reported in previous studies in association with pain sensitivity, involve both cognitive and emotional variables that are interrelated. Despite its pervasive impact on quality of life and the significant burden that it places on healthcare systems, a standardized and reliable method for assessing pain sensitivity that encompasses all these aspects is still lacking. This is especially true when considering the trait dimension of pain experience rather than the state dimension that is associated with the current and real state of pain. This gap poses a significant challenge for researchers and clinicians, as it hampers the accurate diagnosis of pain-related conditions, the management of chronic pain, and the evaluation of therapeutic interventions. Notable limitations [11] were reported for current methods of pain assessment, including self-report scales like the Visual Analog Scale (VAS) and the Numeric Rating Scale (NRS), as well as observational tools such as the Behavioral Pain Scale (BPS). The self-reported experience of pain is susceptible to variability and potential bias in pain measurement due to individual differences in perception and expression, as well as the physical condition of the subject during the assessment [12]. Furthermore, these tools often fail to capture the multidimensional nature of pain, which encompasses sensory, emotional, and cognitive components [13]. New tools should be developed and tested to provide a consistent metric that could help in measuring the features that may affect our response and management of pain, both acute and chronic. This would facilitate more rigorous research into pain mechanisms and treatments [14]. This approach would not only improve patient outcomes but also facilitate advancement in our understanding of the underlying mechanisms of pain. The development of a reliable and valid measure of pain sensitivity requires an interdisciplinary approach [15]. The present study aims to develop a brief self-report instrument for the preliminary assessment of multifaceted aspects of pain, as well as for the assessment of pain experience reported in non-current pain. Accordingly, we have devised a self-rating instrument, the Pain Multidimensional Questionnaire (Pa-M-QU), which is based on several dimensions of pain. Following an examination of previous studies on pain and pain sensitivity assessment, we identified three main and recurrent dimensions that comprise pain experience [2,3,5], i.e., (i) catastrophizing pain, which is characterized by an exaggeration and dramatization of pain and pain consequences and management, (ii) pain sensitivity, which is define as a trait rather than state of pain, and (iii) pain interferes and coping, which is defined as the aspects that may justify coping with pain and that are useful for describing the individual’s pain experience independently by the state of pain. We selected these aspects because a conceptual model could account for the diverse correlates and the consequences of pain might also be useful in a complex explanatory model in the area of mental health related to pain perception. The instrument was assessed for its reliability and validity. The Pa-M-QU was designed with the specific intention of facilitating a more comprehensive assessment of the multifaceted nature of pain experiences.

## 2. Materials and Methods

### 2.1. Participants

Two independent samples were recruited to conduct exploratory factor analysis (EFA) and confirmatory factor analysis (CFA). A first version of the questionnaire for EFA was spread via a KoboToolbox, v. 2.023, survey to collect data from the Italian-speaking population. Only completed surveys were included in the analyses. Participants could withdraw from the study at any time without providing any justification. The sole criterion for inclusion was that the participants be over the age of 18 years. A sample of 362 respondents was included in the analyses (mean age = 29.36; SD = 12.88; female = 72%). CFA was carried out on a sample of 123 respondents (mean age = 28.0; SD = 13.2; female = 74%) who completed a second survey.

### 2.2. Measures

Demographic Questionnaire. A demographic questionnaire collected information about age, gender, and level of education. Moreover, a survey for CFA, which included preliminary information on the diagnosis of chronic illness and or chronic pain, pharmacological treatment, frequency of nociceptive pain experience, common pain type, and physical extensivity of pain expedite, was collected (see Table 1).

Pain Multidimensional Questionnaire (Pa-M-QU). A focus group comprising clinical psychologists, cognitive psychologists, and neuropsychologist screened 138 items. All items encompass multiple aspects of pain (for a review, see Main, 2016 [16]), with a particular focus on the characteristics of pain sensitivity, pain catastrophizing, and coping with pain. From these items, a pool of 59 items was then selected. Subsequently, according to the hypotheses of this study, the items were divided into three different independent scales, which were subjected to three independent exploratory factor analyses and sensitivity analyses (see Results Section). The participants were instructed to reflect on their own painful experiences and indicate the degree to which they had experienced each of the thoughts or feelings represented by the items when experiencing pain on a 5-point scale from 0 (absolutely false) to 4 (absolutely true). The final version of the questionnaire provided respondents with the following instruction: “The following definitions refer to the condition of physical pain and to experiences or states that may be experienced or felt in life contexts related to pain. Please indicate on a scale from 0 (absolutely false) to 4 (absolutely true) the extent to which each definition represents your individual experience with pain”. For further details on the final version of the questionnaire, refer to the Results Section.

Pain catastrophizing. The term “pain catastrophizing” is used to describe a cognitive and emotional process whereby individuals experience a heightened sense of distress and anxiety in response to pain. The Italian validation of the Pain Catastrophizing Scale (PCS) [17] was used to evaluate the extent of catastrophic thinking about pain and to assess the convergent validity of the new questionnaire. The PCS is composed of 13 items on a five-point Likert scale (ranging from 0 = “not at all” to 4 = “all the time”), developed for use with both clinical and non-clinical populations. The Italian version of the scale, which assesses the helplessness, rumination, and magnification dimensions of catastrophizing, has satisfactory psychometric properties, consistent with those observed in the original version (Cronbach’s α range for 0.56 to 0.89). The total score ranges from 0 to 52, with higher scores indicating higher levels of pain catastrophizing (Cronbach’s α = 0.92).

### 2.3. Procedure

A two-step data collection process was employed. In the first phase of the study, data were collected to perform an exploratory analysis (EFA) via an online survey (using KoboToolbox), which was disseminated on the main social media platforms to all potential respondents aged 18 and above. A second independent sample of volunteers completed a second survey, employing similar strategies, to confirm the psychometric aspects via confirmatory factor analysis (CFA). To ensure anonymity, no personal information that could allow the identification of participants was collected. The procedure was approved by the ethical committee of the Department of Dynamic and Clinical Psychology and Health Studies (“Sapienza” University of Rome; protocol number: 0001168, 21 August 2019) and conformed to the Helsinki Declaration.

### 2.4. Data Analysis

A three-step analysis was conducted to test the reliability and validity of the instrument. The focus group identified specific items for each scale of the questionnaire and included them in the appropriate scales. Then, different strategies were adopted to select the most reliable items for each scale [18]: (a) removing items that allow improving Cronbach’s alpha, (b) removing any item with an item–total correlation lower than 0.20, and (c) ranking the remaining items with the removal of one of the similar items if they correlate highly (>0.75). This strategy enabled us to maximize homogeneity. Following the removal of items through item analysis, psychometric properties were tested. The factorial structure of the scales was examined using both exploratory (EFA) and confirmatory factor analyses (CFA) for each scale proposed by the focus group (i.e., catastrophizing of pain, pain sensitivity, coping with pain). These analyses were conducted on two independent samples. Oblimin rotation was used in EFAs because there was no reason to assume that the extracted factors were orthogonal. A scree plot was used to determine the number of extracted factors. However, each factor with an eigenvalue equal to or higher than 1 was considered. The number of factors suggested by the EFAs was then cross-validated in the CFA. Maximum likelihood (ML) estimation was employed in the CFA. Goodness-of-fit was assessed using chi-square, the Comparative Fit Index (CFI), the Tucker–Lewis Index (TLI) and the Standardized Root Mean Square Residual (SRMR) and Root Mean Square Error of Approximation (RMSEA) indices. The cut-off criteria for determining the fit indices were based on Kline’s suggestions [19].

Pearson’s r correlations were calculated to describe the relationship between some of the sample’s characteristics (age, years of education) and the Pa-M-QU global score. Moreover, the convergent validity of the construct was evaluated through the correlations with the PCS. Jamovi and open-source software R (R-Core, 2018 [20]) were used to perform statistical analyses in the current study.

## 3. Results

### 3.1. Items Analysis

From the pool of 59 items identified by the focus group, 14 were included in the Catastrophizing scale, 21 in the Pain Sensitivity scale, and 17 in the Pain Interferes and Coping scale. Then, the analysis of reliability indicated that 35 items should be removed. The final scale consists of 24 items.

### 3.2. Reliability and Exploratory Factor Analysis

Cronbach’s alpha coefficients for all scales suggested good reliability (see Table 1). An examination of the scree plots and the percentages of variance accounted for revealed the presence of a single factor for each scale. The KMO and Bartlett’s test statistics for each scale indicated that the data were suitable for factor analytic procedures [21] (Table 1).

### 3.3. Reliability and Confirmatory Factor Analysis

The CFA confirmed the three monofactorial structures with optimal fit indices (see Table 2).

### 3.4. Convergent Validity and Inter-Correlations

Person’s linear correlations were carried out between the scores of each scale of the Pa-M-QU and the total score of the PCS and the three monofactorial structures positively correlated with each other and with the PCS (see Table 3).

### 3.5. Characteristics of the Sample and Consideration of the Scale

Table 4 shows the main characteristics of the sample considering pain-related information and experience. Table 5 shows quantitative results for each scale and distribution of the scores across the participants.

## 4. Discussion

Our study was conducted within a framework that emphasized the multifaceted nature of pain. The development of the Pa-M-QU was centered on the attempt to assess this complexity by screening some of the multiple components that may influence the self-reported experience of pain, i.e., catastrophizing of pain, pain sensitivity, and pain interferes and coping. The tool demonstrated good reliability in a three-monofactorial structure. Moreover, the fit indices of the CFA yielded highly satisfactory results, indicating a notable degree of stability of the questionnaire in the analysis of the questionnaire pain components in the general Italian population.

### 4.1. Pain Sensitivity

Regarding sensitivity, our scale is not intended to quantify localized pain perception, which previous works have assessed in overt states of pain or experimental conditions through self-rating measures of pain (for a review, see [11]). Instead, in accordance with other authors, our scale aims to improve understanding of general pain sensitivity. Two existing questionnaires, the Central Sensitization Inventory (CSI) [22] and the Pain Sensitivity Questionnaire (PSQ) [10], have been previously developed to assist in the identification of pain sensitivity. The authors considered these questionnaires to be relevant for both clinical purposes in chronic pain conditions and for the assessment of pain intensity in healthy subjects experiencing pain-induced conditions. The PSQ addresses different body sites and pain modalities, requesting respondents to imagine pain. The CSI is a self-report measure designed to identify patients with symptoms that may be related to Central Sensitization (e.g., fibromyalgia and temporomandibular disorders). Given that pain sensitivity is not unidimensional [23], we included a scale of pain sensitivity in a questionnaire that globally considers different facets of pain experience. Further, this scale encompasses both bodily (e.g., Item 4: “When I happen to hit a part of my body against the edge of a hard surface (such as a table) the pain is hardly tolerable for me”; Item 5: “I can’t eat foods that are too hot because this results in pain”) and central dimensions of pain sensitivity (e.g., Item 1: “People who are close to me often tell me that I am too sensitive to pain”; Item 3:“It is very easy for me to feel pain”). The present study thus would suggest the utility of the Pa-M-QU for a rapid assessment of general pain sensitivity. Potential future applications include experimental pain sensitivity assessments in healthy subjects and the prediction of acute pain (e.g., post-operative pain) or chronic pain disorders. It is also relevant to consider pain sensitivity from a cognitive perspective. Indeed, recent findings suggest an increasing association between cognitive aspects and pain sensitivity, pain threshold, and tolerance [24,25,26]. Cognitive factors, such as attention, expectation, and emotional regulation, can significantly influence pain perception and modulation. A comprehensive assessment of pain sensitivity requires understanding the interplay between cognitive, social, and physiological factors. This understanding can inform the development of more effective pain management interventions. Further studies can adopt this screening tool to detect cognitive and affective components of pain further.

### 4.2. Catastrophizing Pain

The term “catastrophizing” is used to describe a maladaptive cognitive style involving the occurrence of exaggerated negative thoughts and emotions during actual or anticipated instances of pain. In previous studies, different foci were directed toward the exaggeration or dramatization of threat or pain [27] or pain-related worry and fear, with an inability to divert attention from pain [28]. From these definitions of pain catastrophizing, research focused on developing reliable self-report instruments for the assessment of this phenomenon. For example, the Coping Strategies Questionnaire (CSQ) by Rosentiel and Keefe included a subscale for helplessness and pessimism in pain contexts [29], which was expanded by Sullivan et al. [30] with the Pain Catastrophizing Scale (PCS), assessing helplessness, rumination, and magnification associated with pain experience. Also, our purpose was to improve the measurement of pain catastrophizing, including it in a screening scale for evaluating this construct in the general population incorporating multiple aspects of pain catastrophizing (e.g., Item 3: “When I feel pain, I can’t help but think about it.”; Item 5: “When I feel pain, I am always afraid that this will increase”; and Item 6: “When I feel pain, I feel that everything is useless and that the pain is about to overwhelm me.”). This is crucial because accurately evaluating catastrophizing can inform treatment strategies and improve patient outcomes. By understanding how and to what degree individuals magnify pain threats and experience helplessness, clinicians can adapt their interventions to mitigate these maladaptive thoughts and behaviors, which may ultimately reduce the overall burden of chronic pain. Furthermore, the Pa-M-QU correlates with measures of catastrophizing (i.e., PCS score), thus providing a viable and brief alternative.

### 4.3. Pain Interferes and Coping

Effective pain management is of crucial importance for improving patient outcomes and quality of life. An understanding of the ways in which individuals manage pain and cope with it is essential and requires a focus on cognitive, emotional, and behavioral strategies. Several well-established questionnaires are used for the evaluation of pain management techniques, each with its particular strengths and limitations. For example, the Coping Strategies Questionnaire (CSQ; [29]) includes subscales for evaluating cognitive and behavioral coping strategies, such as diverting attention, reinterpreting pain sensations, and using positive self-statements. Also, the Pain Self-Efficacy Questionnaire (PSEQ; [31]) measures the confidence of individuals in performing activities despite their pain.

In this regard, the PSEQ shows the impact of self-efficacy on pain management. Nevertheless, despite the usefulness of these questionnaires, several critical aspects highlight the need for developing additional tools. One significant limitation is the inadequate consideration of the social and contextual factors that influence pain management. Pain management is a multifaceted process affected by social support, cultural background, and environmental context. Comprehensive assessment tools that capture these dimensions are essential for holistic pain management [32]. Another critical aspect is the dynamic nature of pain management strategies. Individuals may change their coping strategies over time in response to treatment, psychological state, and social interactions. The development of tools capable of tracking changes in pain management strategies longitudinally and providing real-time data would be helpful in pain studies [33]. The experience of pain may fluctuate depending on the activities in question. For example, an individual with chronic back pain may experience an exacerbation of symptoms following a day spent seated at a desk and a subsequent improvement following a yoga class. A significant limitation of many pain assessments is that they only reflect the intensity of pain experienced or reacted to during the test session without providing any insight into the subject’s overall pain experience at the time of the test. In this sense, a functional perspective can be the analysis of individual response to different representations of pain experience from early and less severe forms of pain to generalized response to pain (e.g., Item 1: “When I experience even minimal pain, this prevents me from carrying out normal activities of daily living (e.g., doing household chores, going to work, studying, etc.).”; Item 3: “When I experience pain, I can’t help but focus on what hurts.”; and Item 6: “When I feel pain, I cannot sleep”).

### 4.4. General Considerations and Limitations

The three-monofactorial structure of this questionnaire, including three independent but correlated scales assessing different features of pain with a limited number of items, represents a key objective achieved by this study. The aim of this study was to develop a brief but reliable screening tool for pain experience, including more than just a single domain, such as pain sensitivity or pain catastrophizing. As previous studies have indicated, a multidimensional analysis of how individuals feel and react to pain is relevant. This analysis should be conducted independently of the current presence of pain, as it is particularly relevant in clinical practice. Although this tool needs further studies to verify its reliability in clinical practice as a possible screening questionnaire for pain sensitivity, and to evaluate a possible cut-off, which is relevant to cover a limitation that emerged in this work, we found promising evidence on pain’s multifactorial nature.

The Pa-M-QU, especially as suggested by the CFA, is a valid tool for this purpose but some limitations should be reported. Firstly, the monofactorial structure of the scales included in the questionnaire precludes the possibility of identifying a global score of pain experience on which the scales may converge. However, this is not possible because pain is a multifaceted phenomenon with many definitions and expressions, which makes it challenging to measure its experience as a global and univocal dimension. Another limitation of this study is represented by the absence of a sample of individuals experiencing clinical pain, specifically those with chronic pain conditions. In fact, despite 61 percent of respondents reporting frequent experience of nociceptive pain, only one respondent indicated a diagnosis of a chronic pain condition. The inclusion of such a sample would have provided valuable insights when compared with a group of individuals without chronic pain. This is because the components investigated by the scales may interact bidirectionally with the chronic pain diagnosis. This could result in the exacerbation and perpetuation of the catastrophizing of pain, alteration in its sensitivity, and an influence on its management [34,35]. Further studies should provide the inter-rater and test–retest reliability of the Pa-M-QU in multiple clinical populations. Finally, pain may be a causal factor in the development of negative emotional reactions, such as transient or chronic anger, depression, loneliness, and anxiety [36,37,38], also in the absence of a chronic clinical condition. This is evidenced by studies that have demonstrated that such emotional states can modify the subjective experience of pain, amplifying the processing of pain signals. Therefore, further studies could also consider these variables in relation to Pa-M-QU scores.

## 5. Conclusions

The Pa-M-QU has demonstrated a significant and adequate fit index for evaluating some aspects of pain in healthy subjects. If its reliability is confirmed in further study, the Pa-M-QU would provide a rapid, straightforward, and non-invasive approach to assessment with good psychometric propriety. The evidence reported in this work may be extended in the analysis of acute pain and in the investigation of pain sensitivity as a potential risk factor for the development of chronic pain disorders. In line with the cognitive–behavioral model of pain treatment, aspects such as pain experience, coping mechanisms, and other psychological components may highly affect outcomes. Future research should focus on evaluating to extend the research on the Pa-M-QU as an alternative or supplementary method to traditional experimental pain assessments in these contexts. Finally, the goal is to develop a reliable instrument for measuring the efficacy of pain interventions in clinical settings.

## Figures and Tables

**Table 1:** Results of EFA and Factor Loading for each item of the scales. Each item is presented in English, with the original Italian version provided in parentheses.

Catastrophizing of Pain		Pain Sensitivity		Pain Interferes and Coping	
KMO = 0.80 Bartlett X^2^ = 322; *p*< 0.0001		KMO = 0.83 Bartlett X^2^ = 408; *p* < 0.0001		KMO = 0.83 Bartlett X^2^ = 532; *p* < 0.0001	
Cronbach’s alpha = 0.70		Cronbach’s alpha = 0.72		Cronbach’s alpha = 0. 77	
	Factor Loading		Factor Loading		Factor Loading
1. I can tolerate physical pain with relative ease. (Il dolore fisico per me è facilmente sopportabile)	0.48	1. People close to me often tell me that I am too sensitive to pain. (Le persone a me vicine spesso mi dicono che sono troppo sensibile al dolore)	0.34	1. When I experience even minimal pain, it prevents me from performing normal activities of daily living (e.g., doing household chores, going to work, studying, etc.). (Quando provo dolore, anche minimo, questo mi impedisce di svolgere le normali attività di vita quotidiana (ad es. svolgere le attività casalinghe, andare al lavoro, studiare, ecc.)	0.37
2. Immediately upon experiencing even the slightest pain, I contact the physician to request a prescription for pain medication. (Appena inizio ad avvertire anche un minimo dolore, contatto subito il medico affinché possa prescrivermi dei farmaci antidolorifici.)	0.47	2. I think I would be able to have a minor medical procedure (such as a few stitches) without worry. (Penso che riuscirei a sottopormi a una piccola operazione (per es. pochi punti di sutura) senza nessuna preoccupazione.	0.57	2. When I feel physical pain, I am forced to stay in bed all day. (Quando inizio a percepire un dolore fisico, sono costretto a stare a letto per tutta la giornata.)	0.59
3. When I feel pain, I am forced to think about it all the time. (Quando provo dolore non riesco a fare a meno di pensarci)	0.40	3. It is very easy for me to feel pain. (È molto facile per me sentire dolore).	0.33	3. When I have pain, my focus is on the pain. (Quando provo dolore non riesco a fare a meno di concentrarmi su ciò che mi fa male)	0.55
4. When I feel pain, I feel that this will never end (Quando provo dolore sento che questo non finirà più)	0.43	4. If I happen to bump a part of my body against the edge of a hard surface (like a table), the pain is hardly sustainable for me. (Quando mi capita di urtare una parte del mio corpo contro il bordo di una superficie dura (ad esempio un tavolino) il dolore è per me difficilmente sostenibile)	0.51	4. When I feel pain, I think I can handle it. (Quando provo dolore, penso di poterlo gestire)	0.67
5. When I feel pain, I am always afraid that this will increase (Quando provo dolore ho sempre paura che questo possa aumentare)	0.53	5. I can’t eat food that’s too hot because it causes me pain. (Non riesco a mangiare cibi troppo caldi perché questo mi crea dolore)	0.57	5. I often think about how pain causes me suffering. (Penso spesso a quanto il dolore mi provochi sofferenza)	0.36
6. When I feel pain, I feel that everything is useless, and the pain is about to overwhelm me (Quando provo dolore sento che tutto sia inutile e che il dolore stia per sopraffarmi)	0.50	6. I experience a lot of pain if I happen to irritate my eyes with soap while taking a bath or shower. (Provo molto dolore se mi capita di irritarmi gli occhi con del sapone mentre mi faccio il bagno o la doccia)	0.47	6. When I feel pain, I cannot concentrate on other activities (Quando provo dolore non riesco a concentrarmi su altre attività)	0.41
7. When I feel pain, it is hard for me to think of anything besides pain. (Quando provo dolore, è difficile per me pensare a qualcosa oltre al dolore)	0.51	7. I feel a lot of pain when I have a muscle cramp. (Provo molto dolore quando ho un crampo muscolare)	0.60	7. When I feel pain, I can’t sleep (Quando provo dolore non riesco a dormire)	0.82
8. When I feel pain, I can‘t think of anything else to overcome it (Quando provo dolore non riesco a pensare ad altro che superarlo)	0.57	8. I experience a lot of pain when I undergo a blood draw (Provo molto dolore quando mi sottopongo a un prelievo ematico)	0.65	8. When I experience pain, I am unable to engage in distracting activities. (Quando provo dolore non riesco a distrarmi)	0.61

Rating scale of the questionnaire was developed on a 5-point scale from 0 (absolutely false) to 4 (absolutely true). KMO: Kaiser–Meyer–Olkin test.

**Table 2:** Results of CFA.

Catastrophizing of Pain	Pain Sensitivity	Pain Interferes and Coping
CFI = 0.93; TLI = 0.90; SRMR = 0.04; RMSA = 0.05 (CI 90% = 0.03–0.07)	CFI = 0.98; TLI = 0.97; SRMR = 0.03; RMSA = 0.04 (CI 90% = 0.001–0.06)	CFI = 0.98; TLI = 0.97; SRMR = 0.03 RMSA = 0.04; (CI 90% = 0.005–0.06)

CFI: Comparative Fit Index; TLI: Tucker–Lewis Index; SRMR: Standardized Root Mean Square Residual; RMSEA: Root Mean Square Error of Approximation.

**Table 3:** Correlations between the Pa-M-QU scales and the PCS.

	Catastrophizing of Pain	Pain Sensitivity	Pain Interferes and Coping
Pain Sensitivity	0.61 **	-	
Coping with Pain	0.77 **	0.67 **	-
PCS	0.63 **	0.62 **	0.78 **

** *p*< 0.001. PCS: Pain Catastrophizing Scale.

**Table 4:** Characteristics of pain in the sample for CFA.

	*n* (%)
*Chronic Pathologies*	
Yes	15 (12.2)
No	108 (87.8)
*Chronic Pain Diseases*	
Yes	1 (0.8)
No	122 (99.2)
Pharmacological Treatment for Pain	
Yes	12 (9.8)
No	111 (90.2)
Frequency of Experiencing Pain	
Almost Never	2 (1.6)
Sometimes	37 (30.1)
Frequently	75 (61.0)
Mostly	9 (7.3)

**Table 5:** Mean and standard deviation of the scales of the Pa-M-QU, according to the items reported by the CFA.

	Catastrophizing of Pain	Pain Sensitivity	Pain Interferes and Coping	PCS
Mean and Std.Dev.	9.30 (4.37)	7.84 (5.04)	8.52 (5.90)	15.04 (9.58)
Scores over 2 Std.Dev	18.04	17.92	20.32	34.2
% of participant overcoming 2 Std.Dev.	4 (5/123)	4 (5/123)	5 (6/123)	

Here, 2/123 (2% of respondents) overcome the average of the sample of 2 Std.Dev. for all the three scales of the Pa-M-QU.

## Data Availability

Data are available by contacting corresponding author.
